# Fractal dimension and lacunarity analysis of mandibular bone on digital panoramic radiographs of tobacco users

**DOI:** 10.34172/joddd.2021.024

**Published:** 2021-05-05

**Authors:** Suman Basavarajappa, Vijayalakshmi Konddajji Ramachandra, Shrawan Kumar

**Affiliations:** ^1^Department of Oral Medicine and Radiology, Government Dental College & Research Institute, Bangalore, India

**Keywords:** Fractals, Lacunarity, Mandible, Panoramic, Radiography, Tobacco

## Abstract

**Background.** This study aimed to evaluate and compare changes in the mandibular trabecular bone pattern using the fractal dimension (FD) and lacunarity analysis in tobacco users with healthy controls.

**Methods.** This study was carried out on digital panoramic radiographs of 225 subjects divided into three groups: smokeless tobacco users (SLTs), smokers, and control (n=75). ImageJ program with FracLac plugin was used to assess the FD and lacunarity of mandibular trabecular bone on the digital panoramic radiographs.

**Results.** The differences in the mean FD values of the study and control groups were statistically significant (*P* < 0.001). Mean FD was lower in the case groups than the control group, with SLTs having the least FD value. A significant difference in lacunarity was noted between SLTs and controls (*P* < 0.001). On the contrary, there was no significant difference in lacunarity between smokers and controls.

**Conclusions.** FD values were lower in tobacco users, suggesting that tobacco users have a less complex trabecular bone pattern than healthy controls. Higher lacunarity values in SLTs indicated a more heterogeneous bone pattern. These findings signify that FD and lacunarity analysis on digital panoramic radiographs can serve as promising predictive tools to assess bone quality for osteoporotic changes in tobacco users, thereby facilitating prompt referral for further management.

## Introduction

Globally, 1.27 billion people are currently using tobacco products, leading to approximately 5.4 million deaths per year.^1^ In India, according to the Global Adult Tobacco Survey (GATS) conducted by the World Health Organization (WHO) in 2016‒2017, 28.6% of Indians habitually use tobacco products (42.4% of males and 14.2% of females). It is alarming that the Indian population is more inclined to use smokeless tobacco (21.4% of adults) than smoking tobacco (10.7% of adults).^2^

Tobacco use is a cause of concern for various systemic diseases like certain types of cancer, cardiovascular diseases, respiratory ailments, and bone diseases like osteoporosis. Osteoporosis, defined as an asymptomatic systemic bone disease, is characterized by low bone mass and micro-architectural deterioration of bone tissue, with a consequent increase in bone fragility and susceptibility to fracture.^3^ Osteoporotic fracture is a significant public health problem globally^4^ and imposes a significant financial and social burden. Therefore, utmost attention is required for the prevention, early diagnosis, and prompt treatment of osteoporosis.

Osteoporosis is known to cause greater relative loss of trabecular than of cortical bone. Changes in the bone trabecular pattern can be characterized by some measures, including the area of the bony plates, the circumference of the trabeculae, the number of bony and marrow regions, the thickness of trabeculae, trabecular spacing, and osseous fractal dimension (FD). Dual-energy x-ray absorptiometry (DEXA) scan is considered the gold standard for the detection of osteoporosis. Yet, it is an expensive technique with limited availability^5^ and variability in the results due to the instrument’s model and operation mode, thereby compromising its utility extensively. This disadvantage can be reasonably managed by employing a digital panoramic radiograph, which is a convenient, cost-effective screening tool with wide availability and low radiation dose. With the advent of digital imaging, several researchers have tried to collect more information from digital images with digital image processing and analysis techniques.^6^ Digital panoramic imaging, with its practical processing capacities, offers the prospect of refined qualitative and quantitative analyses of bone density and architecture, thereby facilitating the early detection of osteoporotic changes with appropriately applied image analysis algorithms.^7^

The introduction of fractal analysis for the evaluation of complex structures in biology and medicine has been recognized by several researchers. The osseous fractal analysis is a useful mathematical technique for describing and analyzing the intricate structural patterns of trabecular bone using two statistical measures known as the FD and lacunarity. FD characterizes the structural complexity numerically with an increasing number, indicating an increase in complexity. FD of trabecular bone has been associated with bone strength. It has been used for evaluating complex interconnections of alveolar cancellous bone on dental images enabling the identification of subjects with or without osteoporosis. In the presence of similar FD values but with a different appearance visually, lacunarity, another method of further classifying these, can be employed. Lacunarity describes the spatial distribution of lacunae or gaps and is a measure of how the fractals fill the space.^8-11^ The trabecular bone shows fractal characteristics, such as self-similarity and lack of well-defined scale due to its branched structure. Therefore, fractal geometrical applications can be used to define the complex structure of trabecular bone.^12^

The effects of tobacco on the mandible identified by the fractal analysis, such as FD and lacunarity on digital panoramic radiographs, might help in the early recognition of osteoporotic changes and facilitate prompt referral for bone density assessment and further management. Besides, there is hardly any scientific literature available for such analysis in tobacco users. Hence, this study aimed to assess the FD and lacunarity on digital panoramic radiographs of mandibular trabecular bone in tobacco users and compare it with healthy controls.

## Methods

### Study groups

This cross-sectional study was conducted in the Department of Oral Medicine and Radiology on 225 subjects from September 2017 to August 2018 with the approval of the Institutional Ethics Committee. The guidelines of the Helsinki Declaration were followed in this study. The study evaluated 75 smokeless tobacco users (SLTs) and 75 smokers, both with a habit duration of more than a year. Seventy-five age-and-sex matched healthy controls were also included with no harmful habits, aged 20‒50 years, who voluntarily consented to participate. Subjects with a previous history of jaw fracture, reconstructive surgery, orthodontic treatment, edentulism, history of pathological lesions of the jaws/surgery, periapical lesions in the region of interest (ROI), para-functional habits like clenching and bruxism, systemic diseases or chronic illnesses, skeletal, renal, and hepatic disorders, history of anti-resorptive drug therapy, long-term corticosteroid therapy and drugs affecting the bone metabolism, and women with surgically induced menopause or subjected to hormonal replacement therapy were excluded.

### Image acquisition

Digital panoramic radiographs of the selected 225 subjects were taken by the same operator using a digital panoramic and cephalometric system (Kodak Dental Systems CS-9000C, France) with standard exposure parameters of 70 kVp, 10 mA, and 14 seconds. Standard head positioning with adequate and appropriate radiation safety protocol was followed.

### Fractal dimension and lacunarity analysis

**Figure 1:**
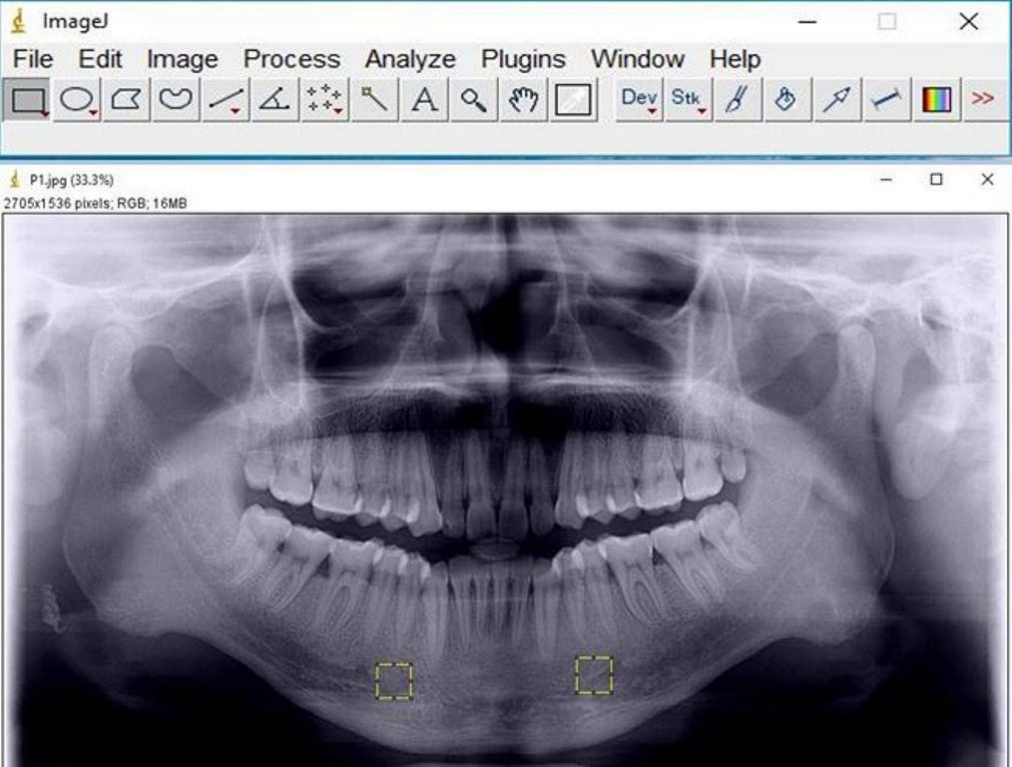
Selection of the region of interest (ROI) bilaterally on a panoramic radiograph in ImageJ software.

**Figure 2:**
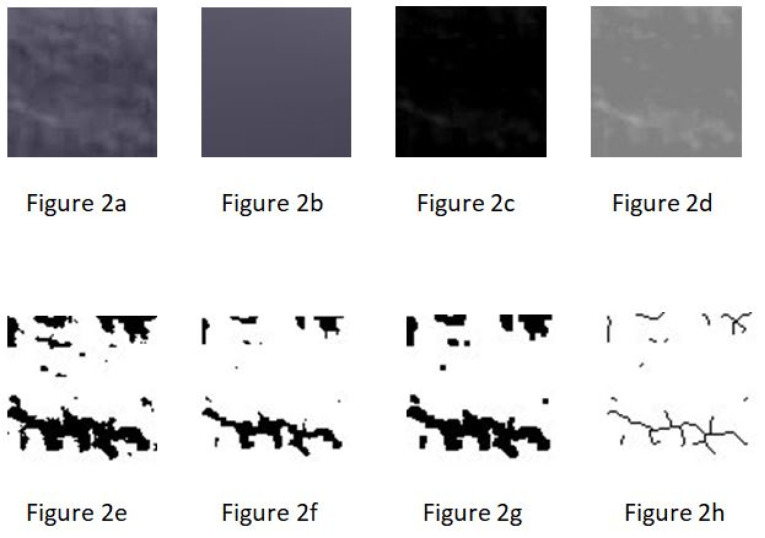
Image processing procedures. (a) The duplicated region of interest (81*81 pixels). (b) Gaussian blurred image (diameter 35 pixels). (c) Blurred image subtracted from the original image d). Addition of 128 to the result. (e) A binary image. (f) Erosion process. (g) Dilated image. (h) skeletonized image.^5^

Digital panoramic radiographs of all the patients were in TIFF format, around 200 kb in size. The images were randomly shuffled and viewed on a 17-inch LED monitor with a resolution of 1024×768, 32 bits, 2 GB RAM, 1.67 GHz processor with landscape orientation under subdued lighting. The ROI, a square-shaped box of 81* 81-pixel size, was selected manually in the mandible bilaterally anterior to the mental foramen and below the root apices^8^ in each radiograph to prevent the interference of hyoid bone and the effect of anatomical structures, such as mental foramen, mandibular nerve canal, lamina dura and tooth roots, on the analysis (Figure 1). The selected regions of interest were processed using the method designed by White and Rudolph (Figure 2a-2h).^5^ The ROIs were duplicated and then blurred by a Gaussian filter with a diameter of 35 pixels. The resulting heavily blurred image was then subtracted from the original image. Bone marrow spaces and trabeculae were discriminated from each other by adding a 128-grey value to each pixel location. The image was then binarized to outline bone marrow spaces and trabeculae. The noise of the resulting image was eliminated with erosion, and the outlines of the structures were emphasized using dilation. The image was then inverted to make the trabeculae black and bone marrow spaces white. The skeletal structure indicated the bone marrow pattern, and the non-skeletal structure represented bone marrow in the skeletonized binary image. After skeletonization, the ROIs were prepared for evaluation of FD and lacunarity.^5^

FD was calculated using the box-counting method in ImageJ 1.51k program. First, the images were converted using square grids of equal size widths of 2, 3, 4, 6, 8, 12, 16, 32, and 64 pixels. The resulting number of tiles was plotted against the total number of tiles in a double logarithmic scale. FD was calculated from the slope of the line fitted on the data points.

**Figure 3:**
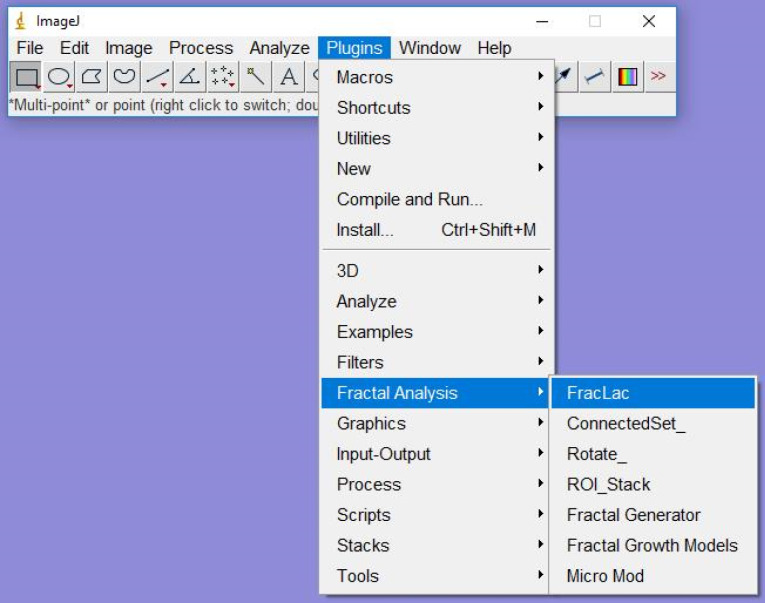
Showing FracLac plugin option in ImageJ application used for the calculation of lacunarity.

Lacunarity was calculated using a plugin named FracLac (Figure 3). FracLac plugin has options of the box counting method and sliding box method for lacunarity analysis. We used the box counting method of the FracLac plugin for the calculation of lacunarity. ImageJ is a public domain software that facilitates image processing and analysis (ImageJ, National Institutes of Health, Bethesda, MD). FD and lacunarity were calculated bilaterally for each radiograph, and the mean of these two values was considered for the statistical analysis.

### Statistical analysis

The data were analyzed using SPSS 22.0 (IBM Corp., New York). One-way ANOVA was used to compare mean FDs and mean lacunarity between the three groups. To determine which specific group’s means were different, Tukey’s HSD tests were used for multiple comparisons of mean difference in FD and lacunarity between the three groups. Pearson’s correlation was used to analyze the correlation between FD, lacunarity, and habit characteristics (frequency and duration) in each group. The results were reported as means ± standard deviations and a level of *P* < 0.05 was deemed statistically significant.

## Results

The control group’s mean age was 37.8±8.2 years, with 37.8±8.4 and 35.2±6.4 years in the SLTs and smoker groups, respectively. The number of males and females with smokeless tobacco habits was 66 (88%) and 9 (12%), respectively. However, the smoker group consisted of males only. The gender distribution in the control group comprised 71 (94.7%) males and 4 (5.3%) females. The frequency of using smokeless tobacco was 2‒11 times, with an average of 4.6 times daily. The duration of smokeless tobacco use was 1‒25 years, with an average of 8.6 years. The frequency of smoking was 2‒24 times, with an average of 10 times daily. The duration of smoking was 3‒30 years, with an average of 9.1 years.

Table 1 shows the comparisons of mean FD and mean lacunarity between the groups. Table 2 shows specific comparisons of mean differences in FD and lacunarity between the study and control groups. While the mean FD of the control group was 1.5043±0.0597, the mean FD values for SLTs and smokers were 1.4432±0.0619 and 1.4781±0.0505, respectively. The differences in the mean FD values of the study and control groups were significant (*P* < 0.001). Mean FD was lower in study groups than the control group, with SLTs having the least FD value. The mean lacunarity of 0.2789±0.0326 was noted for SLTs, whereas it was 0.2579±0.0366 for smokers. The mean lacunarity for the control group was 0.2592±0.0300. A statistically significant difference was noted between the study groups and controls (*P* < 0.001) for lacunarity. Comparing mean differences in FD and lacunarity between SLTs and controls revealed a statistically significant difference (*P* < 0.001). A statistically significant difference was also noted in mean FD between smokers and controls (*P* < 0.02); however, there was no significant difference for mean lacunarity between these groups. A statistically significant difference was noted between SLTs and smokers for mean FD and mean lacunarity, with SLTs having a lower FD and higher lacunarity than smokers.

**Table 1:** Comparison of means and standard deviations of variables among the groups

**Variables**	**Groups**	**N**	**Mean**	**SD**	**Minimum**	**Maximum**	***P*** ** value**
Fractal dimension	SLTs	75	1.4432	0.0619	1.316	1.55	< 0.001*
	Smokers	75	1.4781	0.0505	1.337	1.558	
	Control	75	1.5043	0.0597	1.346	1.616	
Lacunarity	SLTs	75	0.2789	0.0326	0.219	0.379	< 0.001*
	Smokers	75	0.2579	0.0366	0.191	0.342	
	Control	75	0.2592	0.0300	0.209	0.322	

SD, standard deviation; SLTs, smokeless tobacco users. * Significant.

**Table 2:** Multiple comparisons of mean differences in fractal dimension (FD) and lacunarity between the groups

**Variables**	**Group(I)**	**Group (J)**	**Mean difference (I-J)**	**95% CI of the difference**		***P*** ** value**
				**Lower**	**Upper**	
Fractal dimension	SLTs	Smokers	-0.0349	-0.0571	-0.0127	0.001*
		Control	-0.0611	-0.0833	-0.0389	< 0.001*
	Smokers	Control	-0.0262	-0.0484	-0.0040	0.02*
Lacunarity	SLTs	Smokers	0.0210	0.0082	0.0338	< 0.001*
		Control	0.0197	0.0069	0.0325	0.001*
	Smokers	Control	-0.0013	-0.0141	0.0115	0.97

CI, confidence interval; SLTs: smokeless tobacco users. *Significant.

Table 3 shows the correlation between FD, lacunarity, and habit characteristics (frequency and duration) in each group. There was a very weak negative correlation between the frequency of smokeless tobacco use and the mean FD. However, a moderate negative correlation with statistical significance was noted between smokeless tobacco use and FD duration. A weak negative correlation with statistical significance was also noted between mean FD and frequency and duration of smoking. A very weak correlation without any statistical significance was noted between mean lacunarity and frequency and duration of smokeless tobacco use. However, a weak positive correlation with statistical significance was noted between mean lacunarity and frequency and duration of smoking.

**Table 3:** Correlation analysis of fractal dimension (FD) and lacunarity values of tobacco users with tobacco habit characteristics

**Variables**	**Groups**	**Habit characteristics**	**r**	***P*** ** value**
Fractal Dimension	SLTs	Frequency	-0.20	0.09
		Duration	-0.54	< 0.001*
	Smokers	Frequency	-0.39	0.001*
		Duration	-0.38	0.001*
Lacunarity	SLTs	Frequency	0.13	0.28
		Duration	-0.04	0.75
	Smokers	Frequency	0.30	0.009*
		Duration	0.37	0.001*

R, correlation; SLTs, smokeless tobacco users *Significant.

## Discussion

Osteoporosis might pose a significant threat to human health and the world economy in the coming times. Among the toxic substances involved in the etiology of osteoporosis, tobacco plays a significant role, and it is considered a potentially modifiable risk factor. Wong et al have suggested the possible mechanism by which smoking can affect bone health by increased hepatic metabolisms of vitamin D metabolites and impaired calcium absorption.^4^ Decreased serum calcium due to the interference of parathormone action with renal tubules in smokers has also been noted.^13^ Decreased vitamin D levels, increased free radicals, and oxidative stress in smokers are associated with bone resorption. Various studies have provided evidence that smoking affects the balance of the naturally occurring processes of bone resorption and bone formation, resulting in low bone mineral density.^3^ It has also been reported that cigarette smoke extract inhibits in vitro differentiation of osteoprogenitor cells to osteoblast-like cells.^14^

Inhibition of osteoblastic metabolism due to smokeless tobacco extracts and the effect on osteoblasts’ viability as a result of inhibited immune reactions due to areca nut extracts in betel quid has been demonstrated in many in vitro studies. The role of areca nut extracts on the viability and gene expression of alkaline phosphatase, receptor activator of nuclear factor kappa-β ligand (RANKL), and osteoprotegerin in human osteoblasts were suggested by Abbas et al.^3^ According to Quandt et al, increased bone loss with smokeless tobacco use has been observed to be a detrimental effect of smokeless tobacco on the bone. Also, it has been reported that smokeless tobacco provides higher amounts of nicotine to users than does cigarette smoking. Nicotine induces vasoconstriction, low tissue oxygen tension, and tissue ischemia.^15^ All these together can adversely affect bone. The mandible is highly sensitive to alterations in the body bone mass, and a definite correlation between mandibular and skeletal (vertebral) bone densities has been observed in several studies.^16^

Like other body tissues, the mandible is said to undergo a gradual decrease in mineralized bone throughout life. Recent literature states that an association exists between osteoporosis and jaw bone loss, and mandibular morphology alters due to osteoporosis.^17^ The dentist often sees many adult subjects in the dental clinic and might recommend a panoramic radiograph as part of the diagnostic protocol. Digital panoramic radiography is the best imaging modality with a low radiation dose. It is used extensively for broad coverage of jawbones and serves as a cost-effective tool for studying osseous changes in the mandible. Several studies have used densitometric and radiomorphometric measurements to assess bone in quantitative and qualitative parameters. Digital panoramic radiography has the processing potential to analyze bone density and architecture qualitatively and quantitatively and can detect osteoporotic changes early with suitably applied image analysis algorithms.^7^

An image’s texture is a collection of many small components, and the analysis of texture can be carried out either statistically or structurally. The basic characteristic of fractal geometry is self-similarity. Fractal analysis has been applied in various fields, from applied mathematics to chemistry and physics to biology and medicine. Fractal analysis is a useful technique in the quantification of complex structures. Several researchers have agreed that it is a useful, inexpensive, reliable, and easily applied method to analyze bone pattern.

Link et al^18^ investigated the trabecular structure of human vertebral and femoral bone. They used high-resolution magnetic resonance and computed tomography images combined with texture analysis using morphometric measures and box-counting FD and compared these techniques with bone mineral density. It was concluded that texture analysis using FD might provide additional information to analyze bone strength and quality.^18^ Various studies have been carried out to differentiate subjects with and without osteoporosis using fractal analysis on dental radiographs.^6,8,19^ Doyle et al^20^ suggested the possibility of detecting osteoporosis with fractal analysis of dental radiographs. They reported a preliminary study where FD of mandibular radiographs of postmenopausal women was higher than that of premenopausal women.^20^ Southard et al^21^ in an in vitro study examined radiographic FD changes in a decalcified human alveolar bone process. They found that the average FD value decreased and stated that the radiographic FD holds promise for detecting osteoporosis. The results of the present study are consistent with these results.^21^

It has been reported that fractal analysis can be used to identify the bone trabecular pattern and define the complex shape and structural pattern. FD is a quantitative method to measure these complex shapes and patterns. It describes how an object occupies space and represents the complexity of the object, with a higher FD value indicating more complexity. Fractal images, however, have a limited range of self-similarity.^6^ Different fractal sets might share the same FD and have extremely different textures.^6^ For discriminating these similar textures, the term lacunarity, coined by Mandlebrot, is used to further classify fractals and textures with the same FD but a different visual appearance. It was developed to define the property of fractals and can be used to describe the spatial distribution of real data sets.^6^ This is an advantage of lacunarity over FD. It is a measure of how the fractal fills the space and is related to the distribution of gap sizes. According to Dougherty and Henebry, lacunarity plots explicitly characterize an image’s spatial organization, including the average size of any structural sub-unit(s) within an image, making them potentially useful in representing the trabecular thinning and perforation of vertebral trabecular bone associated with osteoporosis.^22^ The geometric objects having low lacunarity are homogenous as all the gap sizes are the same, whereas objects with high lacunarity are heterogeneous.^6^ In this study, FD and lacunarity were evaluated on digital panoramic radiographs to assess mandibular trabecular bone structure in SLTs and smokers.

Yasar and Akgünlü,^6^ in a study on direct digital periapical images of the mandibular posterior region in dentate and edentulous subjects, assessed FD and lacunarity and stated that dentate and edentulous areas have different trabecular bone textures. According to them, the differences in bone structures can be discriminated using FD and lacunarity. In the present study, FD and lacunarity values of controls, SLTs, and smokers were compared, and the FD values of tobacco users were significantly lower. A significant increase in lacunarity was noted in SLTs compared to controls. SLTs had a lower FD and higher lacunarity, suggesting a less complex and heterogeneous trabecular structure than controls. Smokers had lower FD values than controls, indicating a decrease in the complexity of trabecular structure in smokers. It was also noted that SLTs had a lower FD and higher lacunarity than smokers, indicating decreased complexity and heterogeneous pattern of bone in SLTs than smokers.

Although there is no research in the medical literature to which this study can be compared directly, several studies have been carried out on FD analysis in osteoporosis and similar diseases related to bone structure. Gumussoy et al^12^ carried out a survey on 25 chronic renal failure subjects and evaluated FD on panoramic radiographs and found that FD values in subjects were lower than those in controls. A similar trend of decrease in FD for tobacco users was noted in this study. Ergun et al^19^ carried out a fractal analysis on panoramic radiographs of a patient with primary hyperparathyroidism and concluded that FD values of the patient decreased before parathyroidectomy and showed osteoporotic bone characteristics.^19^ Akin to this, in the present study, tobacco users demonstrated lower FD values.

Kiel et al^23^ reported an independent association between smoking during adulthood and bone mineral density, suggesting the possibility of peak bone mass reduction due to smoking.^23^ It has been reported that bone is influenced by dose (frequency) and the duration of smoking, and increased smoking exposure can lead to a more significant loss in bone mineral density.^24^ In the present study, there was a negative correlation between FD and frequency and duration of smoking, suggesting an increase in trabecular bone loss with an increase in frequency and duration of smoking.

Several researchers^20,25,26^ have reported that FD increases in diseases, leading to osteoporotic effects on bone structure. However, others^8,12,21,27^ concluded that FD value decreases, consistent with this study. Thus, FD values obtained in various studies are contradictory. The conflicting results can be attributed to various factors, such as exposure time and image resolution, anatomical variations, fractal analysis techniques, and different methods for calculating FD.^8^ According to Veenland et al^28^ and Geraetes and Van Der Stelt,^29^ FD could be affected by the noise produced during the imaging process. Therefore, a study on FD values should be carefully designed to obtain a more accurate result.

All the study subjects suspected of osteoporotic changes were referred for further evaluation and management. There are some limitations to this study. Bone mineral density values obtained by the gold standard method, DEXA, could have been compared with FD and lacunarity for a better result. Another limitation of the study is that the panoramic radiographs are two-dimensional representations of three-dimensional structures. CBCT would have provided more precise information, but it was not used in this study as it is not a regularly used method and due to its high radiation dose and cost for the subject. Studies with a larger sample size will also facilitate a better amount of FD in tobacco users as the bone structure might be anatomically different in different individuals.

## Conclusion

The present study demonstrated that the FD values in the mandible on digital panoramic radiographs decreased in the SLTs and smokers. An increase in lacunarity was found in SLTs only. These data suggest that SLTs have less complex and heterogeneous trabecular bone pattern and texture than controls. Smokers showed a lower FD, indicating a less complex trabecular bone pattern. Hence, FD and lacunarity can discriminate the texture differences of trabecular bone in tobacco users. Furthermore, it can be used as a practical, non-invasive method to assess osteoporotic changes in tobacco users during a routine examination, thereby facilitating prompt referral for further management.

## Authors’ Contributions

SB contributed to developing the concept of the study, preparation of the manuscript, and supervision of the study. VR contributed to proofreading and editing the manuscript, and SK performed the observations in the study and preparation of the manuscript.

## Acknowledgments

None.

## Funding

Not applicable.

## Competing Interests

The authors declare no competing interests with regards to the authorship and/or publication of this article.

## Ethics Approval

This research was approved by the Institutional Ethical Committee of the Government Dental College and Research Institute, Bangalore, India with reference no. GDCRI/ACM/PG/Ph.D/5/2017-18, dated 29/11/2017.
